# Investigating the functional connectivity between central glucagon-like peptide-1 (GLP-1) and glutamatergic signaling: a systematic review

**DOI:** 10.1017/S1092852926100844

**Published:** 2026-01-29

**Authors:** Sabrina Wong, Gia Han Le, Kayla Teopiz, Roger S. McIntyre

**Affiliations:** 1 Department of Pharmacology & Toxicology, University of Toronto, Canada; 2 Mood Disorders Psychopharmacology Unit, University Health Network, Canada; 3 Brain and Cognition Discovery Foundation, Canada; 4 Institute of Medical Science, University of Toronto, Canada; 5 Department of Psychiatry, University of Toronto, Canada

**Keywords:** Glucagon-like peptide-1, GLP-1, glutamatergic system, glutamatergic modulators, NMDA, AMPA

## Abstract

Glutamatergic neurons represent 40% of neurons in the human central nervous system. Glutamate accounts for approximately 90% of all excitatory neurotransmitters. Previous research reports the presence of glucagon-like peptide-1 (GLP-1) receptors on neurons that produce glutamate. Herein, we aim to evaluate whether GLP-1 receptor agonists’ (GLP-1 RAs) modulate glutamatergic signaling and whether GLP-1 RAs’ anti-obesity effects are mediated through the glutamatergic system. We conducted a systematic review of extant literature published on PubMed, Ovid and Scopus databases from inception to March, 2025. Identified studies were screened independently by two reviewers (S.W. and G.H.L.) using the Covidence platform. We sought to include in vitro, in vivo, and human clinical studies. A total of 31 studies were identified as meeting eligibility for an inclusion in this review. No human studies were identified. Across the included preclinical and pharmacologic studies, GLP-1 RAs were associated with increased glutamate release, NMDA/AMPA receptor activation and increased release of neurotrophic factors associated with neurogenesis, neurodifferentiation, and synaptic plasticity. In addition, GLP-1 RA-induced suppression of food intake was reported to be dependent on AMPA, but not NMDA, receptor signaling. The effect of GLP-1 RAs on feeding behavior is mediated via central glutamatergic signaling. A comprehensive mechanistic framework mediating GLP-1 RA activity implicates crosstalk between GLP-1 and ionotropic glutamate receptors. The aforementioned trends instantiate a need to evaluate the therapeutic efficacy of GLP-1 RAs for disparate neuropsychiatric disorders. Conducting target engagement studies of GLP-1 RAs with the glutamatergic system in humans is a future research vista.

## Introduction

Glucagon-like peptide-1 receptor agonists (GLP-1 RAs) are a class of glucose-lowering agents that are approved by the US Food and Drug Administration for the treatment of type 2 diabetes mellitus (T2DM) and obesity, lowering the risk of major adverse cardiovascular events, as well as lowering the risk of worsening kidney disease, kidney failure, and death due to cardiovascular disease in adults with type 2 diabetes and chronic kidney disease.^1^ GLP-1 RAs mimic the effects of endogenous GLP-1, which is an endocrine hormone produced in the L-cells of the intestine.^2^ Various metabolic effects have been associated with GLP-1, including stimulation of insulin secretion, inhibition of glucagon secretion, slowing gastric emptying, and increasing satiety.^2^ In addition to peripheral metabolic effects associated with GLP-1 RAs, extant literature reports GLP-1 RAs have effects within the central nervous system including improvements in cognitive and reward function, reducing the severity of the disparate domains of suicidality as well as protecting against neurodegeneration.^3^^–^^7^ While the putative mechanism of action of GLP-1 RAs mainly focuses on agonism of the GLP-1 receptor, the downstream effects of GLP-1 receptor activation as well as potential off-target effects of GLP-1 RAs remain incompletely understood. Moreover, while GLP-1 RAs’ anti-obesity mechanism of action mainly focuses on effects in the periphery, GLP-1 receptors have been reported to be present in multiple brain regions including, but not limited to, the hypothalamus, amygdala and the hippocampus.^8^ Research of GLP-1 RAs has also extended toward indications in mental disorders (ie, major depressive disorder [MDD], substance use disorder), which instantiates a need to further evaluate the effects of GLP-1 in the brain.^9^

Notably, glutamate is the most widely expressed excitatory neurotransmitter in the brain with glutamatergic neurons being expressed throughout the brain and spinal cord.^10^ Glutamate signaling and its regulation is crucial for neurodevelopment, learning and memory, and overall brain function.^10^^,^^11^ In addition, the glutamatergic system has been implicated in a number of diseases, including endocrine, neurodegenerative, and mood disorders.^12^ Furthermore, the glutamate system also regulates food intake and appetite regulation.^13^^,^^14^

Replicated study results indicate GLP-1 receptors are present on glutamatergic neurons.^7^^,^^15^^,^^16^ Specifically, preliminary evidence reports that activation of GLP-1 receptors on the glutamatergic neurons increases glutamate release to regulate autonomic and metabolic functions including food intake suppression.^16^ In addition to overlapping physiological effects of the glutamatergic and GLP-1 systems, the foregoing observations suggest the presence of functional connectivity between the two signaling systems. Herein, we sought to determine whether GLP-1 RA signaling involves activity at the canonical glutamatergic signaling pathway. Secondarily, we aimed to evaluate whether GLP-1 RAs have effects on feeding behavior or weight change that are mediated by effects on the glutamatergic system.

## Methods

### Databases and search strategy

This systematic review was conducted in accordance with the 2020 Preferred Reporting Items for Systematic Reviews and Meta-Analyses.^17^ To evaluate the effects of GLP-1 RAs on glutamatergic signaling, we performed a systematic search on PubMed, Ovid (ie, Medline, Embase, APA PsychInfo, AMED, JBI EBP), and Scopus databases from inception to March 5, 2025. An additional manual search was conducted on Google Scholar and through citation searching to ensure all possible studies were captured. For details on the search strings used for each database, see Supplementary Table S1.

### Screening and eligibility criteria

Studies that were identified in the database search were screened independently by two reviewers (S.W. and G.H.L.) using the Covidence platform. Studies were first screened by title and abstract. For studies to proceed to the full-text screening stage, individual studies must have been considered relevant by at least one of the reviewers. Studies that were screened by their full text were screened against the eligibility criteria. Studies to be included must (1) be a primary study (ie, randomized controlled trial, open-label trial, intervention trial, animal study, in vitro study), (2) investigate the effects of a GLP-1 receptor agonist on glutamatergic and/or NMDA/AMPA receptor activity in vitro and/or in vivo, (3) for human participants, they must be 18–64 years of age, inclusive, (4) be published or translatable to English, and (5) have full-text availability. Studies were excluded if they met at least one of the following exclusion criteria: (1) non-primary research (e.g., review articles, commentaries, letters to the editor, dissertations, protocols, published abstracts), (2) case reports or case series, (3) does not use a GLP-1 receptor agonist as an intervention, and (4) does not report on glutamatergic or NMDA/AMPA receptor activity. Studies were not excluded based on medical diagnosis of human participants or for the type of animal model being employed. Studies that were included must be unanimously agreed upon by both reviewers. Any discrepancies were resolved through discussion.

### Data extraction

Data was extracted from the included studies by one reviewer (S.W.). The data extraction stage was conducted using a piloted data extraction table. Data to be extracted were established a priori and included the following: (1) Author(s) and publication date, (2) study design (ie, in vitro, animal, clinical), (3) sample size, (4) sample characteristics (eg, animal model, genotype, species), (5) GLP-1 RA investigated and dosing regimen, (6) outcome measures, and (7) main results.

### Risk of bias analysis

Included studies were analyzed for risk of bias based on the study design utilized. Animal studies were evaluated for their methodological quality and risk of bias using the SYRCLE’s risk of bias tool.^18^ As there is currently no validated risk of bias tool to analyze in vitro studies, we were unable to evaluate the included in vitro studies. Risk of bias assessments were conducted independently by two reviewers (S.W. and G.H.L.). Any differences in ratings were resolved through discussion.

## Results

### Search results and component study characteristics

**Figure 1.**
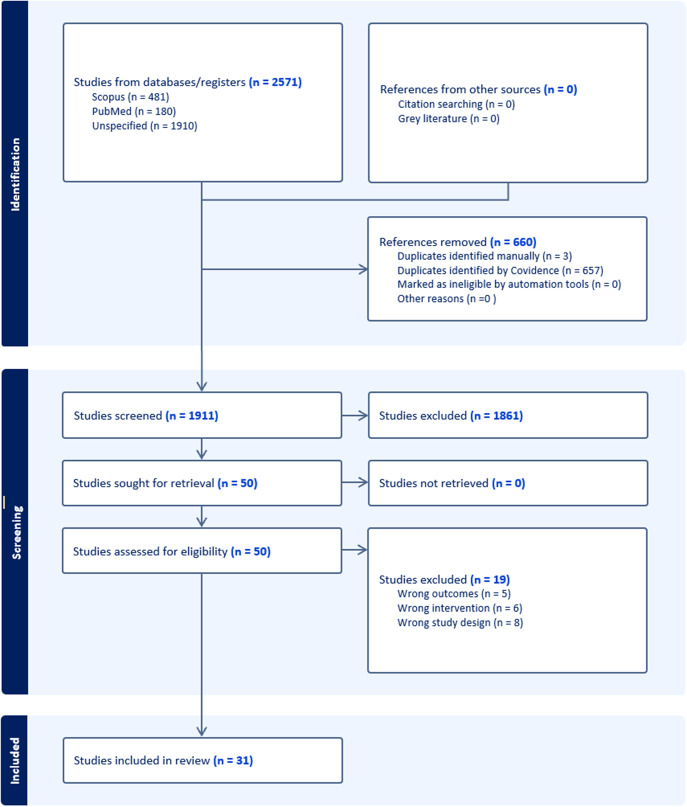
Flow Diagram of Study Screening and Inclusion Process.

The search resulted in the identification of 2574 articles. Following the automatic removal of 660 duplicates, 1914 studies underwent title and abstract screening. A total of 50 studies were considered relevant and underwent full-text screening. Of the 50 studies, a total of 31 studies were considered eligible for inclusion in this review (*n* = 31) For full details on the study screening process, see Figure 1.

Of the 31 studies, 11 studies exclusively utilized in vitro methods, 11 studies exclusively utilized in vivo methods, and 9 studies utilized both in vitro and in vivo methods. We did not identify any human studies that evaluated the effects of GLP-1 RAs on glutamatergic signaling. From the included studies, the investigated GLP-1 RAs included liraglutide, dulaglutide, exendin-4/exenatide and GLP-1(7–36) amide. For details pertaining to the characteristics of each included study, refer to Table 1.

**Table 1.** Study Characteristics of Included Component Studies

Study	Study design	Sample size and/or characteristics	GLP-1 agonist/intervention	Outcome measures	Main results
Abdelwahed et al.^19^	Animal study	36 adult male Wistar albino rats	Exendin–4 3 ug/kg subcutaneously for 30 d	Gene and protein expression of BDNF	Diabetic mice had downregulated BDNF gene (88.18% decrease) and protein (66.15% decrease) expression compared to the negative control and the exendin–4 control group. Exendin–4 significantly upregulated BDNF gene (376.92% increase) and protein (115.13% increase) expression compared to untreated diabetic mice (p < 0.001).
Adams et al.^20^	Animal study	*Glp1r1*-flox, *vGAT*-Cre, *vGlut2*-Cre, *Glp1r*-Cre, and *L10-*GFP reporter mice 10 mice per sex and genotype	Liraglutide 400 ug/kg subcutaneous	GABAergic and glutamatergic neuronal activity, short- and long-term feeding effects	Liraglutide significantly increased Fos + cells in CeA, IPBN, bed nucleus of the stria terminalis, caudal nucleus of the solitary tract, and area postrema. There was no effect in the lateral septum, paraventricular thalamus, PVH, arcuate, ventromedial hypothalamus, lateral dorsal tegmental nucleus or rostral NTS. CeA (93.6 +/− 1.2%) and BNST (88.9 +/− 2.7%) were mostly GABAergic and were *vGAT-*GFP+. In the glutamatergic lPBN, 95.4 +/− 1.1% of Fos + neurons were *vGlut2*-GFP+. The cNTS and AP were heterogeneous in *vGlut2I- and vGAT-*GFP. In the AP, 45.4+/−3.3% of Fos + cells were *vGAT*-GFP+ and 93.2+/− 1.5% were *vGlut2*-GFP+. In *vGlut2* and *vGAT* deficient mice, liraglutide significantly decreased 24 hours food intake compared to the control in the mice that lack *vGAT* and attenuated anorectic effect in mice that lacked *vGlut2.* In obese mice, *Glp1r*-flox control and *vGAT* mice showed a decrease in food intake following liraglutide treatment, but not in *vGlut2* mice. Fos activation in all of the previously activated brain regions was absent in the *vGlut* lacking mice. Fos activation was highest in the IPBN (28.6 +/− 2.2%) and AP (84.3 +/− 1.3%), indicating that glutamatergic neurons in these regions are critical for GLP–1RA activity.
Babic et al.^21^	In vitro study	Whole cell patch clamp recordings on pancreas-projecting neurons 116 pancreas-projecting neurons from 55 rats	Exendin–4 0.45 nmol injection	Group II and III mGluR inhibitory and excitatory postsynaptic currents	Exendin–4 was associated with increased miniature inhibitory postsynaptic currents (mIPSCs) (0.79 +/− 0.12 to 1.27 +/− 0.25 events s^−1^, p < 0.05). Similar trends were observed for APDC treatment (mGluR agonist). For neurons that decreased miniature excitatory postsynaptic currents (mEPSCs) frequency, exendin–4 (100 nM) increased the frequency from 2.83 +/− 0.82 to 4.88 +/− 1.79 events/s, which was not observed for L-AP4. Following L-AP4 treatment that increased mEPSC frequency, exendin–4 increased frequency from 2 +/− 0.53 to 3.69 +/− 1.08 events/s, p < 0.05).
Babic et al.^22^	Animal study	Dorsal vagal complex (DVC), mediobasal hypothalamus (MBH) brain tissues from 72 female Sprague Dawley rats	Liraglutide 0.2 mg/kg twice per day subcutaneously for 6 wk	NMDA NR1 and GAD67 expression	Liraglutide had no effect on GAD67 protein expression in the DVC (F_5,40_ = 2.152, p > 0.05) or the MBH (F_5,43_ = 2.056, p > 0.5). There was also no effect on NR1 protein expression in the DVC (F_5,42_ = 0.493, p > 0.05) or MBH (F_5,41_ = 0.489, p > 0.05).
Bojanowska and Stempniak^23^	In vitro study	Male Wistar rats	tGLP–1 (GLP–1 (7–36) amide)	Vasopressin and oxytocin release via NMDA and non-NMDA receptors	tGLP–1 significantly increased vasopressin and oxytocin release, but none of the glutamate receptor antagonists (that is KA, AP–5, DNQX) affected baseline release of vasopressin and oxytocin. KA completely blocked tGLP–1-induced vasopressin secretion but not oxytocin release. DNQX did not affect tGLP–1’s effects. AP–5 suppressed tGLP–1-induced AVP release, but not oxytocin secretion.
Bomba et al.^24^	In vitro and Animal study	In vitro: Hippocampal and whole cortex samples from mice In vivo: 22 mice with B6; 129 genetic background	Exenatide 500 ug/kg intraperitoneally, 5 d per week for 2 months	BDNF signaling, spine density,	Exenatide increased phosphorylated CREB and BDNF expression in the hippocampus. In the cortex, exenatide did not affect CREB or TrkB activation, but increased BDNF. Exenatide treatment also increased the levels of phosphorylated ERK5 and PSD95. Neurons treated with exenatide (500 nM) increased dendritic spine density compared to vehicle controls, which were blunted when co-treated with ANA–12 (10 uM), an inhibitor of TrkB.
Eakin et al.^25^	In vitro and Animal study	In vitro: Primary neurons from 18 Sprague–Dawley rats In vivo: 26 male Sprague–Dawley rats	In vitro: Exendin–4100 nM and 1 uM In vivo: exendin–4 21.1 ug/ky/d subcutaneously	Protection against glutamate toxicity (100 mM glutamate)	Glutamate toxicity reduced SH-SY5Y cell viability by 37.5%, which was fully ameliorated by exendin–4 for both doses (p < 0.05). Exendin–4 also blocked the increase in caspase–3 activity seen in the control group. Exendin–4 also reduced the number of neurons undergoing apoptosis in the presence of 10 uM glutamate.
Gateva et al.^26^	Animal study	48 Male ICR mice with streptozotocin-induced diabetes	Liraglutide 0.4 mg/kg once daily, ip for 10 d post streptozotocin induction	Glutamine/glutamate ratio	A significant between group difference was observed F(3,47) = 6.76, p < 0.001, n^2^ = 0.19 wherein liraglutide-treated mice significantly differed from diabetes control mice. Liraglutide mice had significantly greater glutamine/glutamate ratios.
Gilman et al.^27^	In vitro study	Hippocampal cell cultures from Sprague–Dawley rats	GLP–1 (7–36) amide 10 nM	Intracellular calcium concentrations and protection against glutamate excitotoxicity (60 uM)	GLP–1 did not affect basal intracellular calcium concentrations. When glutamate was administered, GLP–1 treated neurons had significantly lower increases in intracellular calcium compared to the negative controls. Peak intracellular calcium response and sustained phase of calcium elevation were reduced in GLP–1-treated neurons. Mean glutamate-induced current densities (peak and steady state) were significantly lower in GLP–1-treated neurons compared to control cells. Both peak and sustained phase of intracellular calcium response to K+ were lower in GLP–1 treated neurons along with the magnitude of the calcium current density. Saline-treated neurons that were exposed to glutamate had a 55% reduction in neuron numbers after 24 hours whereas GLP–1-treated neurons had fewer than 10% of neurons die after 24 hours.
Guan et al.^28^	In vitro and Animal study	Male Sprague–Dawley rats Hippocampal slices from rats	Dulaglutide (0.15, 0.3, 0.6 mg/kg)	Protein and RNA expression of PI3K/Akt/mTOR	Following dulaglutide treatment, PI3K, Akt, and mTOR phosphorylation were increased compared to the other groups. Non-phosphorylated forms showed no differences. The high dose dulaglutide groups showed downregulation of PI3K/Akt/mTOR pathway-related Deptor (mTOR inhibitor) and Pdpk1 (upstream kinase of PKB/C-AKT) and upregulation of ribosome-related Rp117 and LOC100362149, compared to the model group.
Iwai et al.^29^	In vitro and Animal study	14-d old Wistar rats of both sexes Rats were induced to have type–1 juvenile diabetes mellitus (JDM)	GLP–1(7–36) amide administered into lateral ventricular region of brain	AMPA and NMDA receptor activity	Fast excitatory postsynaptic potentiation was significantly decreased in both the control (69.3 4.2%, n = 9) and JDM (84.594 5.9%, n = 6) groups compared with each baseline (100.7 0.9% in control, n = 9; 101.5 1.3% in JDM, n = 6); however, the mean magnitude of LTD in the JDM group was significantly lower than the control group. GLP–1 (100 nM) bath application improved the magnitude of LTD in JDM rats (JDM, 85.1+/− 6.1%, n = 6; JDM + GLP–1, 55.5 +/− 4.8%, n = 5; p < 0.0001). However, there was no effect in non-JDM rats. Under higher stimulus strength, GLP–1 significantly increased I-O relation of fast excitatory postsynaptic potentiation of NMDA responses (JDM, R ^2^ = 0.820; JDM + GLP–1, R ^2^ = 0.891; F_1,107_ = 5.17, p < 0.05). For ifenprodil co-treatment, GLP–1’s effects were antagonized (JDM + GLP–1, R ^2^ = 0.895; JDM + GLP–1 + ifenprodil, R ^2^ = 0.959; F_1,65_ = 10.3, p < 0.01). In normal rats, GLP–1 had no effect on the I-O relation of fast excitatory postsynaptic potentiation of NMDA receptors (Control, R ^2^ = 0.820; GLP–1, R ^2^= 0.792; F_1,43_ = 0.0429, p < 0.05).
Koshal and Kumar^30^	In vitro and Animal study	Male albino mice	Liraglutide 75 and 150 ug/kg dissolved in 0.9% saline	GABA and glutamate concentrations post-mortem	Mice that were kindled with pentylenetetrazole had significantly decreased GABA and increased glutamate in the brain. When mice were pre-treated with liraglutide, it significantly prevented alterations in GABA and glutamate compared to the negative controls.
Kutlu et al.^31^	Animal study	Male Balb/c mice	Liraglutide 300 ug/kg/day intraperitoneally for 45 d MK–801 0.25 mg/kg/day intraperitoneally for 45 d	BDNF/TrkB, CREB protein expression	Liraglutide significantly increased BDNF expression (U = 0.0, p < 0.01, z = −2.8). A significant difference was found between groups in the hippocampus BDNF/Trk-B ratio [H (4, 25) = 19.9 p = 0.001]. BDNF/Trk-B ratio increased significantly in the liraglutide group (U = 0.0 p < 0.01 z = −2.8) compared to the control group. There was a significant difference between the groups in the expression of BDNF in the PFC [H (4, 25) = 22.15 p < 0.001]. A significant decrease in BDNF expression was found in the liraglutide group compared to the control group (U = 0.0 p < 0.01 z = −2.8). A significant difference was found between groups in PFC BDNF/Trk-B ratio [H (4, 25) = 15.86 p < 0.01]. BDNF/Trk-B ratio decreased significantly in the Lira-MK group compared to the liraglutide group (U = 0.0 p < 0.01 z = −2.7).
Larsson et al.^32^	In vitro and Animal study	18 GK rats	Exendin–4 0.1 ug/kg intraperitoneally twice daily for 6 wk	Therapeutic potential of exendin–4 in reversing type 2 diabetes-induced neuropathological changes	Exendin–4 treatment had no effect on the number of glutamic acid decarboxylase–67 (GAD67), calretinin (CR) and parvalbumin (PV)-positive cells in the striatum and neocortex. Exendin–4 significantly increased the density of calbindin (CB)-positive cells in the striatum (90% increase, p < 0.01), but not in the neocortex.
Li et al.^33^	In vitro and Animal study	In vitro: SH-SY5Y cells. Primary cortical neurons obtained from 15 male Sprague–Dawley rats In vivo: Male ICR mice	In vitro: 0, 10^−9^, 10^−8^, 10^−7^, 10^−6^ M liraglutide for 1 h In vivo: Liraglutide 20 ug/kg/day for 7 days	Protection from glutamate excitotoxicity (75 and 100 mM)	Liraglutide significantly protected cells against glutamate excitotoxicity at all concentrations for both glutamate doses. Moreover, higher doses of liraglutide promoted cellular proliferation, specifically 10^−7^ and 10^−6^ M. Liraglutide at 10^−6^ M protected cells from 100 mM glutamate-induced cell death, increased cell viability, and prevented increases in caspase–3. 100 nM liraglutide fully protected neurons from glutamate-induced disruption of plasma membrane integrity.
Li et al.^34^	In vitro study	SH-SY5Y neuroblastoma cells and primary cortical neurons from Sprague Dawley rats (cAMP assay)	Exendin–4 (10, 100, 300, 1000 nM), GLP–1(9–36) (10, 100, 3 001 000 nM)	Cell viability following glutamate excitotoxicity	Following GLP–1(9–36) alone in SH-SY5Y cells, there were no differences in cell viability. When pre-treated with GLP–1(9–36) and then treated with a glutamate challenge (100 mM or 150 mM), GLP–1(9–36) dose-dependently protected the cells with significance being reached at 100 and 1000 nM (1000 nM mitigated 33% cell death at 100 mM glutamate and 100 and 1000 nM with 18% mitigation at 150 mM glutamate).
Liu et al.^35^	Animal study	In vitro: Neuro–2a cells In vivo: Wild-type C57Bl/6 mice	In vitro: Exendin–4 10 nM In vivo: Exendin–4 0.1 ug/200 nL	Excitatory synaptic strength, AMPA trafficking	Exendin–4 significantly increased the amplitude, but not frequency, of spontaneous and miniature EPSCs. Exendin–4 in PVN CRH neurons significantly increased AMPA/NMDA receptor-mediated eEPSCs. In the presence of exendin–4, D-APV and picrotoxin, eEPSCs were significantly increased at +40 mV, suggesting that GluA2-lacking AMPA receptors are involved in mediating GLP–1R signaling. When IEM1460, a GluA2-lacking Ca2+ permeable AMPA receptor blocker was added prior to exendin–4, this completely blocked the effect of exendin–4. GluA1-CT-GFP expressing CRH neurons did not show augmentation of mEPSC amplitudes after exendin–4 augmentation and animals with PVN overexpression of GluA1-CT-GFP had blunted food intake suppression effects of exendin–4. When mice were transfected with Neuro–2a cells, exendin–4 significantly increased membrane expression of GluA1 and increased phosphorylation of S845, but not S831, indicating GluA1 S845 is a specific target of GLP–1 signaling. When mice had knockouts of S845, this blocked the exendin–4-mediated suppression of food intake. Finally H–89 blocked the suppressive effects of exendin–4, indicating that exendin–4’s effects on AMPA receptor trafficking is PKA-dependent.
Mietlicki-Baase et al.^36^	In vitro and Animal study	Adult male Sprague–Dawley rats	Exendin–4 (3 ug/kg), exendin–9 (10 ug)	VTA AMPA/kainate receptors activity on food intake	Significant interactions between CNQX and Ex–4 were observed for cumulative food intake up to 24 hours post-treatment (all ANOVAs F_1,21_ > = 4.89, p = < 0.04). VTA GLP–1R activation reduced food intake in part through glutamatergic AMPA/kainate receptor signaling. However, these effects seemed to take place through decreasing meal size (F_1,21_ > = 6.11, p = < 0.03) and minimally on meal frequency (F_1,21_ > = 4.46, p = < 0.05). For VTA samples pretreated with MK–801 (0.05 ug/100 nl) rather than CNQX, suppression of food intake by Ex–4 was not affected (F_1,7_ = < 6.66, p > 0.05). Therefore, GLP–1R activation in the VTA affects food intake- and meal size through glutamatergic AMPA/kainate, but not NMDA, receptors.
Mietlicki-Baase et al.^37^	Animal study	Adult male Sprague Dawley rats	Exendin–4 (1 uM)	GABAergic medium spiny neuronal (MSN) activity in the nucleus accumbens, NMDA/AMPA on food intake	Exendin–4 increased the frequency of MSN mEPSCs (t = 15.60, p < 0.0001) suggesting presynaptic effects of GLP–1 receptor activation. mEPSc kinetics and amplitude were unaffected. Exendin–4 decreased the PPR of evoked EPSCs (t = 4.31, p < 0.01) to further support GLP–1 receptor’s presynaptic effects. Exendin–4 decreased the frequency of action potential firing of MSNs, which was associated with a significant decrease in resting membrane potential (aCSF = −75.3 ± 1.3 mV, aCSF + Ex–4 = −78.6 ± 1.1 mV; p < 0.02). Exendin–4 (0.05 ug) significantly decreased high fat diet intake at 3,6 and 24 hours post-treatment (F_1,7_ > = 5.88, p < 0.05). CNQX attenuated the effects at 6 and 24 h (main effect F > = 5.88, p < 0.05; interaction effect of CNQX and Ex–4 = 7.64, p < 0.03).
Mora et al.^38^	Animal study	6 male Wistar rats	GLP–1(7–36) amide continuous perfusion for 10 min	Glutamine and glutamate extracellular release	GLP–1(7–36) significantly increased glutamine release that lasted 20 min after the perfusion. The foregoing trend was also replicated in glutamic acid release. Both reached statistical significance after the 8th perfusion sample.
Ohtake et al.^39^	In vitro study	Plasma membrane protein extraction from adult male CD1 mice	Exendin–4 0.2 mg/kg subcutaneously 5 times at 3 h intervals	Synaptic plasticity, membrane protein levels of AMPA GluR1/2 and PSD95	Following exendin–4 treatment, CREB phosphorylation significantly increased in the neocortex compared to the controls (168 +/− 18% of control, p = 0.014). BDNF expression was also significantly increased following exendin–4 (145 +/− 10% of control, p < 0.001). Exendin–4 had no significant effects on GluR1 total protein expression (105 +/−5% of control, p = 0.751); however, GluR1 in the plasma membrane fraction was significantly increased (145 +/− 6% of control, p < 0.001). There were no effects of exendin–4 on AMPA GluR2 levels (plasma membrane: 101 ± 3% of the control, p = 0.913; total protein: 94 ± 7% of the control, p = 0.815) or NR1 of the NMDA receptor (plasma membrane: 105 ± 6% of the control, p = 896; total protein: 109 ± 3% of the control, p = 0.779). Moreover, plasma membrane expression of PSD95 was increased with GluR1 (133 +/− 6%, p = 0.004). TMZ pretreatment significantly reduced GluR1 protein expression at the plasma membrane (135% +/− 8% of control, p = 0.0011). Co-administration prevented the effects of exendin–4 on GluR1 membrane insertion (105 +/− 5% of control, p = 0.5789). With exendin–4 alone, PSD95 (132 +/− 4%, p < 0.0001) and BDNF (136 +/− 9%, p = 0.0076) protein expression were significantly increased. When co-administration with TMZ, suppressed the aforementioned increases (PSD95 exendin–4 + TMZ: 110 ± 6% of the control, p = 0.0026 versus exendin–4 alone; BDNF exendin–4 + TMZ: 91 ± 4% of the control, p = 0.0016 versus exendin–4 alone). No effects of TMZ on CREB phosphorylation were observed. Exendin–4 significantly increased mTOR phosphorylation (131 ± 6% of the control), which was abolished when exendin–4 was co-administered with TMZ (87 +/− 3% of control, p = 0.0005). When rapamycin was administered, this abolished the upregulation of GluR1 protein expression by exendin–4 (103 +/− 3% of control).
Palleria et al.^40^	Animal study	Hippocampal slices from streptozotocin-induced diabetes in male Wistar rats	Liraglutide 300 ug/kg per day subcutaneously in mice	mTOR signaling	Liraglutide upregulated phosphorylation of AKT, AMPK, ERK and p706SK. Streptozotocin caused neurodegeneration with decreased phosphorylation of AKT, AMPK, p70S6K, but not ERK1/2. Liraglutide reduced neuronal death and prevented decreases in p70S6K and AKT phosphorylation to control levels, but hyper-phosphorylated AMPK. There were no effects on ERK1/2.
Park et al.^41^	In vitro study	Hippocampal cell cultures from Sprague–Dawley rats	Liraglutide 1 uM	mTORC1, 4E-BP–1, p70SK levels, mTORC1 and AMPA receptor activity, BDNF expression	NBQX (F_1,12_ = 10.140, p = 0.008) and rapamycin (F_1,12_ = 8.279, p = 0.010) blocked liraglutide-induced increases in BDNF expression. Liraglutide did not increase total dendritic length and spine density in hippocampal cells, but did dose-dependently reverse dexamethasone-induced reductions in total dendritic length (F_3,1596_ = 12.860, p < 0.001). Liraglutide significantly rescued dexamethasone-induced changes in spine density (F_3,156_ = 17.000, p < 0.001). Rapamycin and NBQX inhibited liraglutide induction of total dendritic length and spine density. Liraglutide significantly increased expression of PSD–95 (156% of control, p = 0.020), synapsin I (174% of control, p = 0.001), GluA1 (160% of control, p < 0.001). The aforementioned effects were blocked by both rapamycin and NBQX. The foregoing trend indicates that activation of mTORC1 and AMPA receptors are necessary for liraglutide’s effects on dendritic outgrowth and synaptic plasticity.
Petersen et al.^42^	In vitro and animal study	In vitro: Human plasma and HEK293 cells Mouse studies: *db/db* mice Rat studies: Sprague–Dawley rats In vivo pharmacokinetic studies: 8 male DIO C57BL/6 J mice	GLP–1-MK–801 Disulfide bonded GLP–1 and MK–801 at varying concentrations by assay	NMDA receptor activity, metabolic phenotyping, neuronal effects (that is hypothalamic signaling)	RNA-seq revealed that GLP–1-MK–801 had significant upregulation in glutamatergic transcripts such as *Grin2a, Grin2b, Shisa6, Slc17a7.* Also had upregulation of genes related to synaptic transmission such as postsynaptic density and glutamatergic synapse. When comparing RNA-seq results between GLP–1-MK–801 and semaglutide, there were 150 times the number of transcripts relative to the semaglutide group, supporting that GLP–1-MK–801 enriches functional terms pertaining to glutamatergic signaling and synaptic plasticity.
Rebosio et al.^43^	In vitro study	Purified synaptosomes from C57BL/6 J adult male mice	Exendin–4 (1–100 nM)	Activity of GLP–1 receptors in purified cortical and hippocampal synaptosomes	Treatment with exendin–4 (1–30 nM) during KCl-induced depolarization further increased the release of [H^3^]D-aspartate in cortical synaptosomes (37% increase compared to controls). The aforementioned effects were prevented with 10 nM exendin–3 or 10 nM selective adenylyl cyclase inhibitor, 2′,5′-dideoxyadenosine. In hippocampal synaptosomes, exendin–4 (1–30 nM) significantly enhanced KCl-induced release of [H^3^]D-aspartate (43% increase compared to controls). Immunofluorescence results indicated that 40 +/− 2% glutamatergic nerve terminals express GLP–1 receptors in the cerebral cortex, and 20 +/− 3% in the hippocampus. In GABAergic nerve terminals, GLP–1 receptors co-localized at the cerebral cortex at 17 +/− 4% and at 34 +/− 1% in the hippocampus.
Romano et al.^44^	In vitro study	Hippocampal slices from 40 male Wistar rats	Exendin–4 10 ug/kg/day intraperitoneally for 30 d	NMDA receptor signal transduction	Exendin–4 resulted in a significant downregulation of NMDA-R2A and –2B expression compared to the control rats. There was also a significant increase of tyrosine phosphorylated NMDA–2B subunit with exendin–4 treatment. There were no significant changes in NDMA-R2A phosphorylation with exendin–4 treatment.
Turan et al.^45^	Animal study	40 male Wistar rats	Exenatide 0.5 ug/kg subcutaneously	Ca^2+^/CaMKII, PSD95	72 h of REM SD reduced levels of CaMKII and PSD95 in the hippocampus and prefrontal cortex. Exenatide prevented reductions in CaMKII seen in the REM SD mice but not PSD95 in the hippocampus but not the prefrontal cortex.
Wang et al.^46^	In vitro study	Hippocampal primary cell cultures from male Sprague–Dawley rats	Exendin–4 0.2 nmol intrahippocampal injection	Exendin–4 effects on Abeta1–42, NMDA receptors, CaMKII signaling	Exendin–4 alone did not change long term potentiation; however, pretreatment with exendin–4 inhibited Abeta1–42-induced increase in intracellular calcium concentrations, indicating that exendin–4 activity may be through regulating calcium homeostasis. Administration of AP–5 (50 uM) or nifedipine (10 uM) reduced increases in fluorescence by Abeta1–42 indicating that exendin–4 antagonized Abeta1–42-induced elevation in intracellular calcium concentrations through L-type voltage-dependent calcium channels and NMDA receptors. Pretreatment with exendin–4 before Abeta1–42 increased the number of phosphorylated CaMKIIalpha positive cells in the hippocampus.
Wang et al.^47^	In vitro study	Sagittal cerebellar slices from mice brain	Bath application of GLP–1100 nM	Parallel fiber-Purkinje cell (PF-PC) synaptic transmission using whole-cell patch-clamp recording	GLP–1 increased the amplitudes of N1 and N2 parallel fiber stimulation-evoked EPSCs. Normalized amplitude of N1 was significantly higher than baseline (118.6% +/− 5.9% of baseline, p = 0.026). Application of exendin–9–39 blocked all effects of exendin–4. When exendin–4 was co-administered with 100 nM KT57200, a PKA inhibitor, normalized N1 amplitude. PKA inhibitor, 5 uM PKI, in the presence of GLP–1 significantly increased N1 and N2 amplitudes and normalized N1 amplitude (117.3% ± 5.8% of the baseline; p = 0.031). Following administration of gabazine and TTX to block spontaneous EPSCs and GABAergic inhibitory inputs, GLP–1 increased mEPSC frequency but not amplitude. Co-administration of GLP–1 with exendin–9, mEPSC amplitude and frequency were similar to exendin–9–39 alone. KT5720 significantly decreased mEPSC amplitude and did not significantly change frequency. KT5720 co-administered with GLP–1 had no changes in mEPSC frequency and amplitude compared to KT5720 alone.
Wen et al.^48^	In vitro study	Post-mortem brain slices from Adults male Sprague–Dawley rats with PTZ-induced seizure	Liraglutide 0.5 mg/kg/day for 29 d in live rats	Expression levels of neuronal receptors	Exendin–9–39 significantly decreased GABA_A_Rbeta2/3 expression and increased GluR (GluA1–4, GluN1, GluN2A, and GluN2B) expression. When pretreated with liraglutide, but no exendin–9–39, there was significantly increased GABA_A_Rbeta2/3 expression and decreased GluR expression. Treatment exendin–9–39 with liraglutide did not alter expression of neuronal receptors, indicating blocking of liraglutide’s effects.
Zanotto et al.^49^	Animal study	87 male Wistar rats	Exendin–4 10 ug/kg intraperitoneally	Glutamatergic transmission, glutamate uptake, GluN1 content	There were significant between-group differences in glutamate uptake in hippocampal slices (i.e., diabetic vs sham) (F_2,14_ = 10.84, p = 0.0014). 68.3% increase in glutamatergic metabolism with exendin–4 compared with diabetic rats that received vehicle solution (p = 0.0005). GluN1 subunit of the NMDA receptor was significantly reduced in the hippocampus of diabetic rats; however, following exendin–4 treatment, protein levels were normalized to vehicle levels. Exendin–4 transiently increased glutamate uptake until 24 hours in primary astrocytes and acutely (1 hour) in hippocampal slices. Further evaluated the effects of exendin–4 on MG-impaired glutamatergic transmission. Exendin–4 significantly reversed the reductions in glutamate uptake (F_4,40_ = 7.635, p = 0.0001) and GluN1 content (F_4,19_ = 7.74, p = 0.0007).

### Risk of bias results

From the risk of bias analysis, across all of the included studies, there are multiple sources of potential bias. Specifically, the most common sources of bias included an inadequate description of the randomization and blinding methods. As originally described by Hoojimans et al.^18^ randomization and blinding of assessors is not standard practice for animal studies, which may explain why they are not commonly reported in the component studies. Inadequate blinding of study assessors and animal care staff may indirectly influence behavioral outcomes in the animals or may cause time differences when obtaining outcome measures. Differences in outcome measures, especially with metabolic and neuronal outcomes, may significantly interfere with the observed outcomes. Consequently, these and other potential sources of bias may weaken the certainty and strength of the results reported herein. Moreover, the aforementioned sources of bias may affect our inferences and interpretation of the results. Study-specific ratings for each domain are detailed in Supplementary Table S2. In addition, the overall strength of the body of evidence for each included study was assessed using the GRADE Approach for Preclinical Studies, which is detailed in Supplementary Table S3.

### Effects of GLP-1 RAs on glutamate receptor activity and expression

Twelve of the included studies reported on the effects of GLP-1 RAs on glutamate receptor activity and expression. A study conducted by Adams et al. reports that 400 μg/kg liraglutide in vivo was associated with significant increases in Fos + cells across various brain regions including the central amygdala (CeA), lateral parabrachial nucleus (lPBN), bed nucleus of the stria terminalis (BNST), caudal nucleus of the solitary tract (cNTS), and the area postrema.^20^ Notably, the increase in Fos + cells in the CeA and BNST were mostly GABAergic *vGAT*-GFP^+^ neurons while the lPBN primarily expressed glutamatergic *vGlut2*-GFP^+^ neurons.^20^ The area postrema and the cNTS were heterogeneous with expression of both *vGlut2I*- and *vGAT*-GFP neurons. Furthermore, the aforementioned areas with Fos activation was absent in the *vGlut* deficient mice, indicating that liraglutide directly activates a population of glutamatergic GLP-1 receptor-expressing neurons with subsequent engagement of a neural network that is both glutamatergic and GABAergic to elicit physiological effects.^20^

Administration of GLP-1 RAs in hippocampal cultures derived from Sprague–Dawley rats revealed that GLP-1 does not affect basal intracellular calcium concentrations; however, when neurons were pretreated with GLP-1 prior to glutamate administration, GLP-1 significantly attenuated increases in intracellular calcium compared to negative controls.^27^ Moreover, peak intracellular responses and sustained calcium elevation were reduced following GLP-1 treatment. The aforementioned observation was also accompanied by decreased mean glutamate-induced current densities.^27^

Similarly, GLP-1 RA administration inhibited Aβ1–42-induced increases in intracellular calcium concentrations in hippocampal slices of Sprague Dawley rats.^46^ Administration of AP-5 and nifedipine, N-Methyl-D-Aspartate (NMDA) receptor antagonists, also inhibited Abeta1–42-mediated increases in intracellular calcium concentrations. Taken together, GLP-1 RAs may have cytoprotective effects through modulating intracellular calcium concentrations via L-type voltage-dependent calcium channels and NMDA receptors.

When considering the effects of GLP-1 RAs on ionotropic glutamatergic receptor activity, GLP-1(7–36) amide administered into the lateral ventricular brain region of rats with type 1 juvenile diabetes mellitus increased I-O relation of fast excitatory postsynaptic potentiation of NMDA responses (JDM alone: R^2^ = 0.820; JDM + GLP-1: R^2^ = 0.891; F_1,107_ = 5.17, p < 0.05).^29^ Furthermore, ifenprodil co-treatment antagonized GLP-1(7–36) amide’s effects (JDM + GLP-1: R^2^ = 0.895; JDM + GLP-1 + ifenprodil: R^2^ = 0.959; F1,65 = 10.3, p < 0.01).^29^

A separate study conducted by Mietlicki-Baase et al. reported that exendin-4 was associated with increased medium spiny neuron miniature excitatory postsynaptic current (mEPSC) frequency (t = 15.60, p < 0.0001) without affecting mEPSC kinetics and amplitude.^26^ In addition, exendin-4 decreased the paired-pulse ratio of evoked EPSCs (t = 4.31, p < 0.01) as well as decreased the frequency of action potential firing of medium spiny neurons, which was associated with a decreased resting membrane potential (aCSF = −75.3 ± 1.3 mV, aCSF + Ex-4 = −78.6 ± 1.1 mV; p < 0.02). Therefore, GLP-1 receptor activation also activates medium spiny neurons presynaptically through AMPA/kainate glutamatergic signaling.

When considering the effects of GLP-1 RAs on glutamatergic receptor activity, Babic et al. investigated the effects of GLP-1 RAs specifically on group II and III metabotropic glutamate receptor (mGluR) inhibitory and excitatory postsynaptic currents.^22^ GLP-1 was associated with increased miniature inhibitory postsynaptic currents (0.79 +/− 0.12 to 1.27 +/− 0.25 events s^−1^, p < 0.05), which was similar to what was observed with APDC, a group II mGluR agonist.^22^ With respect to miniature excitatory postsynaptic currents, GLP-1 RAs increased the current frequency from 2.83 +/− 0.82 to 4.88 +/− 1.79 events/s, which differed from L-AP4 treatment that increased mEPSC frequency. The aforementioned findings suggest that both group II and III mGluRs are involved in GLP-1 RAs activity.

Individual GLP-1 RAs differentially affect the activation and expression of NMDA receptor subunits. Specifically, Babic et al. reported that in vivo 0.2 mg/kg liraglutide in Sprague Dawley rats had no effects on GAD67 (F_5,40_ = 2.152, p > 0.05) and NR1 protein expression in the dorsal vagal complex (F_5,42_ = 0.493, p > 0.05) as well as the mediobasal hypothalamus (GAD67: F_5,43_ = 2.056, p > 0.5; NR1: F_5,41_ = 0.489, p > 0.05).^22^ Similarly, Ohtake et al. report that exendin-4 had no effect on NR1 expression (plasma membrane: 105 ± 6% of the control, p = 896; total protein: 109 ± 3% of the control, p = 0.779).^39^ Separately, GLP-1(7–36) amide enhanced the activity of the NR2B subunit of the NMDA receptor but did not alter NR2A or NR2B expression.^29^ In contrast, intraperitoneal administration of exendin-4 10 μg/kg/day in the hippocampal slices of male Wistar rats was associated with significant downregulation of NR2A and -2B expression compared to control rats.^44^ The downregulation observed was accompanied with a significant increase in tyrosine phosphorylated NR2B levels, but not NR2A.^44^

The glutamatergic effects of GLP-1 RAs were further confirmed through RNA sequencing, which revealed that administration of disulfide-bonded GLP-1 and MK-801, a NMDA receptor antagonist, (GLP-1-MK-801) resulted in 1568 unique transcripts along with significant upregulation in glutamatergic transcripts such as *Grin2a, Grin2b, Shisa6*, and *Slc17a7.* ^42^ Moreover, compared to semaglutide and MK-801 alone, GLP-1-MK-801 resulted in greater transcript upregulation and enrichment of functional terms, notably transcripts that participate in glutamatergic signaling and synaptic plasticity.^42^

**Figure 2.**
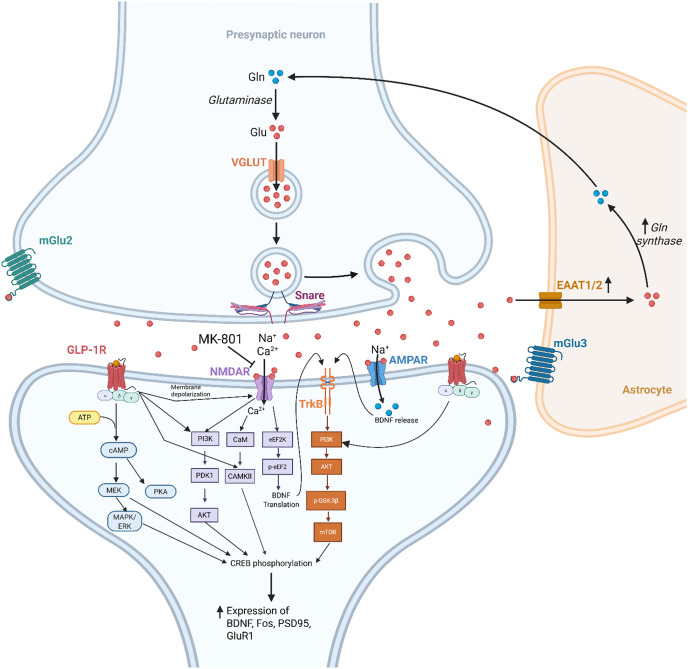
Functional Connectivity Between GLP-1 and Glutamate Receptors.

In addition, GLP-1 RAs were also associated with differential modulation of AMPA receptor expression. GLP-1 RAs differentially affected AMPA GluR expression wherein GluR1 expression in the plasma membrane fraction was significantly increased (145 +/− 6% of control, p < 0.001) but had no effects on GluR2 (plasma membrane: 101 ± 3% of the control, p = 0.913; total protein: 94 ± 7% of the control, p = 0.815).^39^ Plasma membrane expression of PSD95 increased with a commensurate increase in GluR1 expression (133 +/− 6%, p = 0.004) compared to the control group, which suggests that GLP-1 induces GluR1 and PSD95 insertion into the synaptic membrane.^39^ In contrast, Wen et al. reported that in hippocampal slices in seizure-induced Sprague–Dawley rats, exendin9–39, a GLP-1 receptor antagonist, significantly decreased GABA_A_Rbeta2/3 expression and increased GluR expression (ie, GluA1–4, GluN1, GluN2A, GluN2B).^48^ When samples were treated with GLP-1 RA, there was increased GABA_A_Rβ2/3 expression and decreased GluR expression.^48^ Overall, the included studies support that GLP-1 RAs may be associated with increased glutamatergic receptor activity via multiple cellular and molecular mechanisms (Figure 2).

### Effects of GLP-1 RAs on glutamate release, uptake, and glutamate toxicity

We identified four studies that evaluated the effects of GLP-1 RAs on glutamate release and uptake. Specifically, Mora et al. reported that continuous perfusion of GLP-1 RA was significantly associated with increased extracellular glutamine and glutamic acid release.^38^ The aforementioned trend was replicated by Gateva et al. who reported a significantly greater glutamine/glutamate ratio in streptozotocin-induced diabetic mice treated with 0.4 mg/kg liraglutide.^26^ In addition, when mice had pentylenetetrazole-induced epilepsy, there were significant reductions in GABA and increased glutamate, which were prevented through pre-treatment with liraglutide post-mortem.^30^

Separately, Rebosio et al. investigated the effects of GLP-1 RAs on [H^3^]D-aspartate release in purified synaptosomes.^43^ Notably, KCl-induced depolarization was potentiated with GLP-1 RA treatment wherein there was a 37% and 43% increase in [H^3^]D-aspartate release in the cortical and hippocampal synaptosomes, respectively.^43^ Administration of exendin-3, a GLP-1 receptor antagonist, and 2′-5′-dideoxyadenosine both inhibited the observed increased in [H^3^]D-aspartate, suggesting that activation of GLP-1 receptors is associated with increased glutamatergic signaling.

When considering glutamate uptake, Zanotto et al. observed significant between-group differences in hippocampal glutamate uptake between diabetic and control mice (F_2,14_ = 10.84, p = 0.0014) wherein diabetic mice had significantly lower glutamate uptake.^49^ Following GLP-1 RA treatment, there was a significant reversal in glutamate uptake impairment wherein a 68.3% increase in glutamatergic metabolism was observed compared to diabetic rats that received negative control (p = 0.0005). Moreover, GLP-1 RAs transiently increased glutamate uptake up to 24 hours post-treatment in primary astrocytes as well as acutely (ie, 1 hour post-treatment) in hippocampal slices.

Four of the included studies investigated the protective effects of GLP-1 RAs on glutamate-mediated neuronal toxicity. Across all four studies, GLP-1 RAs were significantly associated with protection against glutamate-induced neuronal death. Specifically, at 60 μM glutamate, GLP-1 RA-treated primary hippocampal neurons were reported to have a 10% reduction in neuronal cell viability 24 hours post-treatment, which significantly differed from the 55% reduction observed in the saline-treated neurons.^27^ Similarly at 100 mM glutamate, there was a 37.5% reduction in SH-SY5Y cell viability, which was fully ameliorated by exendin-4 at 1 and 100 uM.^25^ Protection against cell death in SH-SY5Y cells were further replicated with liraglutide and GLP-1(9–36) amide at 75–150 mM.^33^^,^^34^ The foregoing triangulation of evidence indicates that GLP-1 RAs may be associated with increased glutamate metabolism and uptake to protect cells against glutamate excitotoxicity.

### Effects of GLP-1 RAs NMDA/AMPA signal transduction

Nine of the included studies reported on the effects of GLP-1 RAs on NMDA/AMPA signal transduction. Specifically, the expression and activity of secondary messengers including BDNF, mTOR, CREB, PSD95, and CaMKII were evaluated. In addition, one study reported on vasopressin and oxytocin release via NMDA and non-NDMA receptors following GLP-1 administration.^23^

Notably, western blot analysis indicated that GLP-1 RA treatment was associated with increased phosphorylation of CREB, ERK5, PSD95, and BDNF in the hippocampus.^24^ The aforementioned results were also accompanied by increased activation of TrkB in the hippocampus. Furthermore, GLP-1 RA administration in diabetic mice was associated with significant BDNF gene (376.92%) and protein (115.13%) upregulation compared to untreated diabetic mice.^19^ BDNF upregulation was also replicated with liraglutide treatment.^31^^,^^41^ Park et al. report that administration of NBQX, a AMPA receptor antagonist, with liraglutide completely ameliorated liraglutide’s effects on BDNF expression.^41^ When liraglutide was co-administered with MK-801, there were no significant differences in BDNF expression compared to liraglutide alone in the hippocampus (U = 10.0, p = 0.599, z = −0.5) and the prefrontal cortex (U = 0.0, p < 0.01, z = −2.8). The BDNF/TrkB ratio was significantly greater in the liraglutide-treated group compared to control in the hippocampus (U = 0.0, p < 0.01, z = −2.8), which was significantly ameliorated when co-treated with MK-801 (U = 0.0, p < 0.01, z = −2.7).^41^ Similarly in the prefrontal cortex, the liraglutide-treated group had significantly greater BDNF/TrkB ratio compared to the liraglutide and MK-801 co-treated group (U = 0.0, p < 0.01, z = −2.7).^41^ Taken together, GLP-1 RAs may modulate BDNF expression through interaction with the AMPA receptor (Figure 2).

In terms of the expression of second messengers in the PI3K cascade, GLP-1 RAs induced significantly increased phosphorylation of PI3K, Akt, and mTOR compared to the control group.^28^^,^^40^ Notably, GLP-1 RA treatment significantly increased PSD-95 (156% of control, p = 0.020) and synapsin I (174% of control, p = 0.001) expression, which was ameliorated with rapamycin and NBQX.^41^ Furthermore, in Wistar rats that had 72 hours of rapid eye movement sleep deprivation, there were reduced levels of CaMKII and PSD95 in the hippocampus and prefrontal cortex, which was prevented with GLP-1 RA treatment.^45^ Similar trends were observed in rats with Abeta1–42 treated with GLP-1 RAs wherein there was an increase in the number of phosphorylated CaMKIIalpha-positive cells in the hippocampus.^46^ Taken together, GLP-1 RA’s effects on synaptic plasticity and neuroprotective effects may be mediated via mTOR subsequent to AMPA receptor activation (Figure 2).

A study conducted by Bojanowska and Stempniak investigated the effects of GLP-1(7–36) amide (tGLP-1) on vasopressin and oxytocin release through glutamate receptors.^23^ tGLP-1 alone significantly increased both vasopressin and oxytocin release; however, administration of glutamate receptor antagonists, kynurenic acid (KA), AP-5, and DNQX differentially affected vasopressin and oxytocin release. Specifically, KA and AP-5 blocked tGLP-1-induced vasopressin secretion, but not oxytocin, while DNQX did not affect tGLP-1’s effects. As AP-5 abolished tGLP-1’s effects on vasopressin, this suggests that GLP-1’s effects on arginine-vasopressin neurons may be mediated through NMDA receptors.

### Effects of GLP-1 RAs neuron morphology

From the included studies, four investigated the effects of GLP-1 RAs on neuron morphology, specifically dendritic spine density and protection against diabetes-induced neuropathological changes. Notably, in primary hippocampal neurons, GLP-1 RA treatment was associated with increased dendritic spine density compared to vehicle controls.^24^ When an inhibitor of TrkB, 10 μM ANA-12, was administered, GLP-1 RAs’ effects were blunted, suggesting that exenatide may induce increased dendritic spine density through BDNF–TrkB signaling. In rat models for juvenile type 1 diabetes mellitus, GLP-1 RA treatment improved the magnitude of long-term depression (JDM: 85.1+/− 6.1%, n = 6; JDM + GLP-1, 55.5 +/− 4.8%, n = 5; p < 0.0001), which was associated with activation of the NR2B.^29^

In Goto-Kakizaki rats, a model for type 2 diabetes mellitus, GLP-1 RA treatment was associated with a 90% increase in the density of calbindin-positive cells in the striatum.^32^ However, GLP-1 RAs had no effect on the number of glutamic acid decarboxylase-67, calretinin, and parvalbumin-positive cells in the striatum and cortex. Separately, GLP-1 RA pretreatment in Abeta1–42-treated hippocampal primary cell cultures significantly increased phosphorylated CaMKII_alpha_-positive cells indicating that GLP-1 RAs may have neuroprotective effects through glutamate receptor-mediated responses.^46^ Taken together, GLP-1 RAs may have protective effects against neuropathological changes by promoting neurogenesis and synaptic plasticity.

### Effects of functional connectivity between GLP-1 and NMDA/AMPA receptors on food intake

From the included studies, three studies directly evaluated the effects of GLP-1 RAs on food intake and feeding behaviors following glutamatergic modulation. Specifically, Adams et al. measured short- and long-term feeding behaviors in *Glp1r*-flox compared to *vGAT-*, and *vGlut2-*deficient mice following 400 ug/kg liraglutide treatment.^20^ In terms of short-term feeding both in normal and obese mice, liraglutide significantly decreased food intake in the *vGAT*-deficient mice but not in the *vGlut2*-deficient mice. Long-term treatment with liraglutide resulted in a plateau in weight loss effects for all groups.

Separately, a significant interaction effect of GLP-1 RAs and CNQX, an AMPA/kainate receptor antagonist, co-treatment was observed on food intake 3, 6, and 24 hours post-treatment (all ANOVAs F_1,21_ ≥ 4.89, p ≤ 0.04).^36^^,^^37^ Moreover, the foregoing trend indicates that antagonism of AMPA/kainate receptors significantly attenuates food intake suppression effects associated with GLP-1 RA treatment.^26^^,^^37^ The food suppressive effects were attributed to decreased meal size (F_1,21_ ≥ 6.11, p ≤ 0.03) and minimally on meal frequency (F_1,21_ ≥ 4.46, p ≤ 0.05).^36^ When the rats were co-treated with GLP-1 RA and MK-801, GLP-1 RA-induced suppression of food intake was not affected. Co-treatment of GLP-1 RAs and AP-5 also had no effect on food intake or body weight.^37^Taken together, the aforementioned triangulation of evidence indicates that GLP-1 RA-induced suppression of food intake and effects on body weight may be mediated through AMPA/kainate receptor signaling but not NMDA receptors.

## Discussion

Herein, the results of our systematic review suggest that GLP-1 RAs’ ignite a molecular cascade of intracellular events directly. In addition, they also exert their cellular effects via cross functional activity with glutamate neurons and their consequent signal transduction cascades. In general, the included studies suggest that GLP-1 RA administration modulated intracellular calcium concentrations as well as AMPA and NMDA receptor depolarization. Notably, across disparate GLP-1 RAs, AMPA, and NMDA receptor subunit expression was differentially affected. For example, GLP-1 RAs were associated with upregulation of GluR1 with mixed results observed for the effects of GLP-1 RAs on NR1, NR2A, and NR2B. Across the evaluated GLP-1 RAs, there was a consistent trend wherein GLP-1 RA administration was associated with increased AMPA receptor expression and activation as well as increased expression and activity of second messengers associated with neurodifferentiation, neurogenesis and synaptic plasticity. Moreover, GLP-1 RA’s physiological effects on food intake were dependent on AMPA receptor, but not NMDA receptor, activity. Therefore, converging lines of evidence suggest that there is potential functional connectivity between glutamatergic and GLP-1 systems.

In accordance with previously reported studies, GLP-1 RAs administration is associated with increased neurodifferentiation and synaptic plasticity, which potentially mediates GLP-1’s effects on neuroprotection and cognitive and reward function.^3^^,^^50^ Our search results build upon previous studies reporting that GLP-1 RAs are present throughout the glutamatergic system wherein GLP-1 RAs directly interact with ionotropic glutamate receptors to increase the release of BDNF and mTOR activation. The aforementioned triangulation of evidence further supports current research evaluating the implementation of GLP-1 RAs as augmentation treatments for disparate mood and neuropsychiatric disorders that are significantly subserved by metabolic mechanistic underpinnings (eg, MDD, Alzheimer’s disease).^51^ Across various mood and neurocognitive disorders, central insulin resistance and impaired insulin signaling are strongly implicated in the disease and treatment prognosis.^52^^–^^54^ For example, persons with MDD are at a greater risk of developing metabolic syndrome.^55^ Moreover, the directionality of depressive symptom severity and insulin resistance has not been fully established; however, persons with insulin resistance are at greater risk of developing treatment resistant depression, are at increased risk of suicidality and have an overall decreased health-related quality of life.^56^^,^^57^ Therefore, GLP-1 RAs may have broader indications for mood and neuropsychiatric disorders. In addition, replicated evidence indicates that GLP-1 RAs may be effective in the treatment of pain disorders through modulation of inflammatory signaling pathways.^58^ As pain disorders are similarly observed to be subserved by robust glutamatergic disruptions, GLP-1 RAs’ clinical efficacy in the treatment of pain disorders may be partially mediated through the glutamate system. In efforts to explore the aforementioned research area, a research vista that can be conducted in the near future is to design and execute read out demonstrably showing central glutamatergic activity as evidenced by magnetic resonance spectroscopy and electroencephalography.^59^^,^^60^

There are methodological limitations to our systematic review that may affect the inferences and interpretation so the results. Notably, across the included component studies, there were differences in the research methodologies (eg, cell and/or animal model, dosing and frequency of the evaluated GLP-1 RAs, individual GLP-1 RA investigated). Therefore, between-study heterogeneity may limit our ability to determine the degree to which GLP-1 RAs affect glutamatergic signaling across disparate brain regions and how the trends would extrapolate to human populations. Furthermore, due to the aforementioned differences in study methodologies, we could not conduct a quantitative analysis to determine the degree of association or evaluate for the presence of publication bias. Notably within the included studies, several studies utilized models of diabetes and Alzheimer’s disease. Replicated evidence indicates that both diabetes and Alzheimer’s disease are differentially affected by disruptions in the glutamate system, including impaired glutamate uptake, altered receptor expression, and increased excitotoxicity.^61^^–^^63^ These pathological states may amplify or modify the observed effects of GLP-1 RAs, potentially enhancing neuroprotective outcomes. While these models offer valuable insights into disease-specific mechanisms, they may limit the generalizability of findings to other clinical populations.

Finally, we did not identify any human studies reporting on the effects of GLP-1 RAs on glutamatergic signaling. Therefore, our findings may not directly translate to human populations across disparate disease states. Notwithstanding, these findings provide the impetus for future translational research, including early-phase clinical trials or neuroimaging studies, to assess GLP-1 RA-induced glutamatergic modulation. Moreover, given the established clinical use of GLP-1 RAs in metabolic disorders and emerging evidence of neuroprotective and cognitive effects, this mechanistic insight could inform the discovery and development of novel treatments for neuropsychiatric or neurodegenerative conditions characterized by glutamatergic dysfunction.

## Conclusion

Our results indicate that GLP-1 RAs may directly and/or indirectly interact with the glutamate system to mediate their metabolic effects and neuroprotective effects. In addition, GLP-1 RAs may have glutamatergic effects that aid in their therapeutic efficacy for the treatment of disorders associated with metabolic perturbations. Future research should aim to conduct target engagement studies in humans to determine the effects of GLP-1 RAs on glutamatergic signaling and the effects across disparate brain regions. Further investigation of the central metabolic effects of GLP-1 RAs may further inform the research and development of mechanistically informed metabolic and psychotropic agents. In addition, future clinical trials should evaluate whether GLP-1 RAs may work additively or synergistically with co-administered glutamatergic signaling modulators in the treatment and prevention of mental disorders.

## Supporting information

10.1017/S1092852926100844.sm001 Wong et al. supplementary material Wong et al. supplementary material

## Data Availability

This systematic review did not generate or analyze new datasets. All data supporting the findings of this study are derived from previously published studies, which are cited in the reference list.

## Supplementary material

The supplementary material for this article can be found at http://doi.org/10.1017/S1092852926100844.
