# Supplementary material for: Investigating the functional connectivity between central glucagon-like peptide-1 (GLP-1) and glutamatergic signaling: a systematic review
Source: CNS Spectr. 2026 Jan 29;31(1):e4. doi: 10.1017/S1092852926100844 (PMC13076071; doi:10.1017/S1092852926100844)
Supplement: Wong et al. supplementary material [file S1092852926100844sup001.docx]

**Supplementary Material**

**Table S1.** Database Search Strings

| **Database** | **Search String** |
| --- | --- |
| **Pubmed** | (“Glucagon-like Peptide-1” OR “Glucagon-like Peptide-1 Agonist*” OR “GLP-1” OR “GLP-1 Agonist*” OR “Semaglutide” OR “Wegovy” OR “Rybelsus” OR “Ozempic” OR “Dulaglutide” OR “Trulicity” OR “Liraglutide” OR “Victoza” OR “Saxenda” OR “Exenatide” OR “Byetta” OR “Bydureon BCise” OR “Lixisenatide” OR “Adlyxin”) AND (“N-Methyl-D-Aspartate” OR “NMDA” OR “N-Methyl-D-Aspartate Receptor” OR “NMDA Receptor” OR “NMDAR” OR “AMPA” OR “AMPA Receptor” OR “AMPAR” OR “α-amino-3-hydroxy-5-methyl-4-isoxazolepropionic acid” OR “α-amino-3-hydroxy-5-methyl-4-isoxazolepropionic acid receptor” OR “NBQX” OR “Glutamate”) |
| **Ovid** | (“Glucagon-like Peptide-1” OR “Glucagon-like Peptide-1 Agonist*” OR “GLP-1” OR “GLP-1 Agonist*” OR “Semaglutide” OR “Wegovy” OR “Rybelsus” OR “Ozempic” OR “Dulaglutide” OR “Trulicity” OR “Liraglutide” OR “Victoza” OR “Saxenda” OR “Exenatide” OR “Byetta” OR “Bydureon BCise” OR “Lixisenatide” OR “Adlyxin”) AND (“N-Methyl-D-Aspartate” OR “NMDA” OR “N-Methyl-D-Aspartate Receptor” OR “NMDA Receptor” OR “NMDAR” OR “AMPA” OR “AMPA Receptor” OR “AMPAR” OR “alpha-amino-3-hydroxy-5-methyl-4-isoxazolepropionic acid” OR “alpha-amino-3-hydroxy-5-methyl-4-isoxazolepropionic acid receptor” OR “NBQX” OR “Glutamate”) |
| **Scopus** | (“Glucagon-like Peptide-1” OR “Glucagon-like Peptide-1 Agonist*” OR “GLP-1” OR “GLP-1 Agonist*” OR “Semaglutide” OR “Wegovy” OR “Rybelsus” OR “Ozempic” OR “Dulaglutide” OR “Trulicity” OR “Liraglutide” OR “Victoza” OR “Saxenda” OR “Exenatide” OR “Byetta” OR “Bydureon BCise” OR “Lixisenatide” OR “Adlyxin”) AND (“N-Methyl-D-Aspartate” OR “NMDA” OR “N-Methyl-D-Aspartate Receptor” OR “NMDA Receptor” OR “NMDAR” OR “AMPA” OR “AMPA Receptor” OR “AMPAR” OR “α-amino-3-hydroxy-5-methyl-4-isoxazolepropionic acid” OR “α-amino-3-hydroxy-5-methyl-4-isoxazolepropionic acid receptor” OR “NBQX” OR “Glutamate”) |

**Table S2.** Animal Studies Risk of Bias Results

| Study | Item 1 | Item 2 | Item 3 | Item 4 | Item 5 | Item 6 | Item 7 | Item 8 | Item 9 | Item 10 |
| --- | --- | --- | --- | --- | --- | --- | --- | --- | --- | --- |
| Abdelwahed et al. (2018) | Unclear | Yes | Unclear | Yes | Unclear | Unclear | Unclear | Yes | Yes | N/A |
| Adams et al. (2018) | Yes | Yes | Unclear | Yes | Unclear | Unclear | Unclear | Yes | Yes | N/A |
| Babic et al. (2021) | Yes | Yes | Unclear | Unclear | Unclear | Unclear | Unclear | Yes | Yes | N/A |
| Bomba et al. (2018) | Yes | Yes | Unclear | Unclear | Unclear | Unclear | Unclear | Yes | Yes | N/A |
| Eakin et al. (2013) | Yes | Yes | Unclear | Unclear | Unclear | Yes | Unclear | Yes | Yes | N/A |
| Gateva et al. (2024) | Yes | Yes | Yes | Yes | Yes | Unclear | Unclear | Yes | Yes | N/A |
| Guan et al. (2023) | Yes | Yes | Unclear | Yes | Yes | Yes | Yes | Yes | Yes | N/A |
| Iwai et al. (2009) | Unclear | Yes | Unclear | Unclear | Unclear | Unclear | Unclear | Yes | Yes | N/A |
| Koshal and Kumar (2016) | Unclear | Yes | Unclear | Unclear | Unclear | Unclear | Unclear | Yes | Yes | N/A |
| Kutlu et al. (2023) | Yes | Yes | Unclear | Unclear | Unclear | Unclear | Unclear | Yes | Yes | N/A |
| Larsson et al. (2016) | Unclear | Yes | Unclear | Unclear | Yes | Yes | Yes | Yes | Yes | N/A |
| Li et al. (2015) | Unclear | Yes | Unclear | Unclear | Unclear | Unclear | Unclear | Yes | Yes | N/A |
| Liu et al. (2017) | Yes | Yes | Unclear | Yes | Yes | Unclear | Yes | Unclear | Yes | N/A |
| Mietlicki-Baase et al. (2013) | Unclear | Yes | Unclear | Yes | Unclear | Unclear | Unclear | Unclear | Yes | N/A |
| Mietlicki-Baase et al. (2014) | Unclear | Yes | Unclear | Unclear | Unclear | Unclear | Unclear | Unclear | Yes | N/A |
| Mora et al. (1992) | N/A | Yes | N/A | Yes | N/A | N/A | Yes | Yes | Yes | N/A |
| Palleria et al. (2017) | Unclear | Yes | Unclear | Unclear | Unclear | Yes | Unclear | Yes | Yes | N/A |
| Petersen et al. (2024) | Yes | Yes | Yes | Unclear | Yes | Unclear | Yes | Yes | Yes | N/A |
| Turan et al. (2021) | Unclear | Yes | Unclear | Unclear | Unclear | Unclear | Unclear | Yes | Yes | N/A |
| Zanotto et al. (2019) | Yes | Yes | Unclear | Unclear | Unclear | Unclear | Unclear | Yes | Yes | N/A |

Studies were analyzed for risk of bias using the SYRCLE’s Risk of Bias Tool

Item 1 - Sequence generation; Item 2 - Baseline characteristics; Item 3 - Allocation concealment; Item 4 - Random housing; Item 5 - Blinding; Item 6 - Random outcome assessment; Item 7 - Blinding; Item 8 - Incomplete outcome data; Item 9 - Selective outcome reporting; Item 10 - Other sources of bias

**Table S3.** Assessment of the Body of Evidence Using the GRADE Approach for Preclinical Studies

| Study | Outcome Category | Risk of Bias | Inconsistency | Indirectness | Imprecision | Publication Bias | Overall Evidence Rating |
| --- | --- | --- | --- | --- | --- | --- | --- |
| Abdelwahed et al. (2018) | NMDA/AMPA Signal Transduction | Moderate | Low | Low | Low | Not assessed | High |
| Adams et al. (2018) | Glutamate Receptor Activity and Expression | Moderate | Low | Low | Low | Not assessed | High |
| Babic et al. (2012) | Glutamate Receptor Activity and Expression | Moderate | Low | Low | Low | Not assessed | High |
| Babic et al. (2021) | Glutamate Receptor Activity and Expression | Moderate | Low | Low | Low | Not assessed | High |
| Bojanowska and Stempniak (2002) | NMDA/AMPA Signal Transduction | Moderate | Low | Moderate | Moderate | Not assessed | Low |
| Bomba et al. (2018) | NMDA/AMPA Signal Transduction | Moderate | Low | Low | Moderate | Not assessed | Moderate |
| Eakin et al. (2013) | Glutamate Release, Uptake and Glutamate Toxicity | Moderate | Low | Moderate | Low | Not Assessed | Low |
| Gateva et al. (2024) | Glutamate Release, Uptake and Glutamate Toxicity | Low | Low | Low | Low | Not Assessed | High |
| Gilman et al. (2003) | Glutamate Receptor Activity and Expression | Low | Low | Low | Moderate | Not Assessed | Moderate |
| Guan et al. (2023) | NMDA/AMPA Signal Transduction | Low | Moderate | Moderate | Low | Not Assessed | Moderate |
| Iwai et al. (2009) | Glutamate Receptor Activity and Expression | Moderate | Low | Low | Low | Not Assessed | High |
| Koshal and Kumar (2016) | Glutamate Release, Uptake and Glutamate Toxicity | Moderate | Low | Moderate | Moderate | Not Assessed | Low |
| Kutlu et al. (2023) | NMDA/AMPA Signal Transduction | Moderate | Low | Low | Low | Not Assessed | High |
| Larsson et al. (2016) | Glutamate Release, Uptake and Glutamate Toxicity | Low | Moderate | Low | Moderate | Not Assessed | Low |
| Li et al. (2015) | Glutamate Release, Uptake and Glutamate Toxicity | Moderate | Low | Low | Moderate | Not Assessed | Moderate |
| Li et al. (2021) | Glutamate Release, Uptake and Glutamate Toxicity | Moderate | Low | Low | Low | Not Assessed | High |
| Liu et al. (2017) | NMDA/AMPA Signal Transduction | Moderate | Low | Low | Moderate | Not Assessed | Moderate |
| Mietlicki-Baase et al. (2013) | Food Intake | Moderate | Low | Low | Low | Not Assessed | High |
| Mietlicki-Baase et al. (2014) | Glutamate Receptor Activity and Expression | Moderate | Low | Low | Low | Not Assessed | High |
| Mora et al. (1992) | Glutamate Release, Uptake and Glutamate Toxicity | Low | Low | Low | Moderate | Not Assessed | Moderate |
| Ohtake et al. (2014) | NMDA/AMPA Signal Transduction | Moderate | Moderate | Low | Low | Not Assessed | High |
| Palleria et al. (2017) | NMDA/AMPA Signal Transduction | Moderate | Low | Low | Moderate | Not Assessed | Moderate |
| Park et al. (2018) | NMDA/AMPA Signal Transduction | Low | Low | Low | Low | Not Assessed | High |
| Petersen et al. (2024) | Glutamate Receptor Activity and Expression | Low | Low | Low | Moderate | Not Assessed | High |
| Rebosio et al. (2017) | Glutamate Receptor Activity and Expression | Low | Low | Moderate | Low | Not Assessed | Low |
| Romano et al. (2022) | NMDA/AMPA Signal Transduction | Low | Low | Low | Moderate | Not Assessed | Moderate |
| Turan et al. (2021) | NMDA/AMPA Signal Transduction | Moderate | Low | Moderate | Moderate | Not Assessed | Low |
| Wang et al. (2015) | NMDA/AMPA Signal Transduction | Low | Moderate | Moderate | Moderate | Not Assessed | Low |
| Wang et al. (2023) | Glutamate Receptor Activity and Expression | Low | Low | Low | Low | Not Assessed | High |
| Wen et al. (2019) | Glutamate Receptor Activity and Expression | Low | Low | Low | Moderate | Not Assessed | Moderate |
| Zanotto et al. (2019) | Glutamate Receptor Activity and Expression | Moderate | Low | Low | Low | Not Assessed | High |
